# RETRACTION: Risk and Protective Factors Associated With Wound Infection After Neurosurgical Procedures: A Meta‐Analysis

**DOI:** 10.1111/iwj.70452

**Published:** 2025-03-26

## Abstract

**RETRACTION:** HeK. , LiY.‐Y. , LiuH.‐L. , “Risk and Protective Factors Associated With Wound Infection After Neurosurgical Procedures: A Meta‐Analysis,” International Wound Journal 21, no. 2 (2024): e14699, 10.1111/iwj.14699.38346149 PMC10861159

The above article, published online on 12 February 2024, in Wiley Online Library (http://onlinelibrary.wiley.com/), has been retracted by agreement between the journal Editor in Chief, Professor Keith Harding; and John Wiley & Sons Ltd. Following an investigation by the publisher, all parties have concluded that this article was accepted solely on the basis of a compromised peer review process. The editors have therefore decided to retract the article. The authors did not respond to our notice regarding the retraction.

**RETRACTION:** K. He , Y.‐Y. Li , H.‐L. Liu , “Risk and Protective Factors Associated With Wound Infection After Neurosurgical Procedures: A Meta‐Analysis,” International Wound Journal 21, no. 2 (2024): e14699, 10.1111/iwj.14699.38346149 PMC10861159

The above article, published online on 12 February 2024, in Wiley Online Library (http://onlinelibrary.wiley.com/), has been retracted by agreement between the journal Editor in Chief, Professor Keith Harding; and John Wiley & Sons Ltd. Following an investigation by the publisher, all parties have concluded that this article was accepted solely on the basis of a compromised peer review process. The editors have therefore decided to retract the article. The authors did not respond to our notice regarding the retraction.

